# Identification, Characterization, and Epidemiological Analysis of *Lactococcus garvieae* Fish Isolates Obtained in a Period of Eighteen Years

**DOI:** 10.3390/microorganisms13020436

**Published:** 2025-02-17

**Authors:** Ivo Sirakov, Tanya V. Strateva, Vasil Svetoslavov Boyanov, Petya Orozova, Daniel Yordanov, Nikolina Rusenova, Raina Gergova, Svetoslav G. Dimov, Bilyana Sirakova, Vladimir Radosavljević, Liliya Boyanova, Ivan Mitov

**Affiliations:** 1 Department of Medical Microbiology, Faculty of Medicine, Medical University, Sofia, 2 Zdrave, Str., 1431 Sofia, Bulgaria; dr.strateva@abv.bg (T.V.S.); v.boyanov@medfac.mu-sofia.bg (V.S.B.); dr.daniel.yordanov.md@gmail.com (D.Y.); rtgergova@gmail.com (R.G.); liliya.boianova@medfac.mu-sofia.bg (L.B.); 2 National Reference Laboratory for Fish, Mollusc and Crustacean Diseases, National Diagnostic and Research Veterinary Medical Institute “Professor G. Pavlov”, 1000 Sofia, Bulgaria; petyorozova@gmail.com; 3 Department of Veterinary Microbiology, Infectious and Parasitic Diseases, Faculty of Veterinary Medicine, Trakia University, 6000 Stara Zagora, Bulgaria; n_v_n_v@abv.bg; 4 Department of Genetics, Faculty of Biology, University of Sofia ‘St. Kliment Ohridski’, 8 Dragan Tzankov Blvd., 1164 Sofia, Bulgaria; svetoslav@biofac.uni-sofia.bg; 5 Faculty of Dental Medicine, Medical University of Sofia, 1431 Sofia, Bulgaria; biborisova07@gmail.com; 6 “AIPPMPDM”, Ltd., 2800 Sandanski, Bulgaria; 7 National Reference Laboratory for Fish Diseases, Institute of Veterinary Medicine of Serbia, Janisa Janulisa 14, 11000 Belgrade, Serbia; vladimiradosavljevic@yahoo.co.uk

**Keywords:** *Lactococcus garvieae*, salmonid species, PCR, epidemiological typing, phylogenetic analysis

## Abstract

Lactococcosis caused by *Lactococcus garvieae* is a bacterial infection affecting fish with a considerable economic impact. Recently, *L. garvieae* has established itself as an opportunistic pathogen in humans. The aim of the current study was to test classical and molecular-biological methods for the identification of *L. garvieae* and examine antimicrobial susceptibility and capsule production, an important virulence factor. Additionally, tests for differentiation from closely related species, as well as epidemiological typing, were performed. In a period of 18 years (2002–2019), 24 isolates presumptively identified as *L. garvieae* were collected from *Oncorhynchus mykiss* and *Salmo salar* fish obtained either from retail stores or fish farms. In order to confirm the species, optimized PCR-based protocols were used. As a result, 21 of the tested strains were proved to be *L. garvieae* (*n* = 21). The remaining three isolates were *Lactococcus lactis*, *Streptococcus iniae*, and *Enterococcus faecalis*. Epidemiological typing by randomly amplified polymorphic DNA was performed. Except for a single KG+ isolate, all other strains belonged to the European capsular serotype KG−. All *L. garvieae* isolates showed susceptibility to all tested antibiotics with the exception of clindamycin, which was a diagnostic sign. A thorough optimization of diagnostic methods is essential to determining the etiology of specific infections affecting the personnel at risk in fish farms, the food industry, or within the broader community.

## 1. Introduction

Several species belonging to the genus *Lactococcus* including *Lactococcus garvieae*, *Lactococcus plantarum*, *Lactococcus piscium*, and *Lactococcus raffinolactis* have been identified as clinically significant fish pathogens, [1,2]. In recent years, the new species *Lactococcus petauri* and *Lactococcus formosensis*, which are closely related to the *Lactococcus garvieae* species, have been described [3].

*Lactococcus garvieae* (*L. garvieae*, previously known as *Enterococcus seriolicidae*) was initially isolated in 1974 from the Japanese amberjack (*Seriola quinqueradiata*) in Japan [4]. Later, this pathogen has been isolated from mastitis in cows [5]. *Lactococcus garvieae* is a Gram-positive, facultative anaerobe, arranged in pairs or chains. On nutrient media, it grows with small, rounded colonies with smooth borders and a convex profile. It exhibits a catalase-negative reaction and primarily demonstrates alpha hemolysis on blood agar, resembling enterococci and alpha-hemolytic streptococci. As a part of the genus *Lactococcus*, it is able to produce lactic acid from glucose [2,6]. This pathogen has three serological types based on geographical differences: the European capsular serotype, the Japanese capsular serotype, and the non-capsulated serotype from both regions—Europe and Japan. Numerous studies on the pathogenicity of *L. garvieae* have established that encapsulated isolates (KG− serotype) exhibit greater virulence compared to non-encapsulated strains (KG+ serotype) [1].

In routine microbiological practice, lactococci are often misidentified as enterococci (*Enterococcus faecalis* and *Enterococcus faecium*) or streptococci even when commercial biochemical identification panels are used. The precise microbiological diagnosis of *L. garvieae* is challenging because of the close homology of the species belonging to the genus *Lactococcus*. Importantly, *L. garvieae*, *L. petauri*, and *L. formosensis* cannot be discriminated using matrix-assisted laser desorption ionization-time of flight mass spectrometry (MALDI-TOF), 16S sequencing, or biochemical tests [3,7]. In recent years, some relatively new approaches have been developed to identify these species: whole-genome sequencing, intergenic spacer 16S–23S sequencing, *gyrB* gene sequencing, multiplex PCR, and serotype analysis using PCR [7,8,9,10].

*L. garvieae* has emerged as a significant global bacterial pathogen in the aquaculture industry, affecting both freshwater and marine fish, particularly at water temperatures exceeding 15 °C, causing significant economic loss [2,11,12,13]. Lactococcosis has been reported in various aquaculture species, including Nile tilapia (*Oreochromis niloticus*), rainbow trout (*Oncorhynchus mykiss*), yellowtail (*Seriola quinqueradiata*), amberjack (*Seriola dumerili*), cobia (*Rachycentron canadum*), barramundi (*Lates calcarifer*), and catfish (*Pseudoplatystoma* sp.) [13,14,15,16,17,18,19,20]. *L. garvieae* has been isolated from subclinical inflammations in cattle, poultry, pigs, canine, and feline tonsils [21,22,23,24,25,26,27]. Outbreaks among those animals may serve as a significant source with the potential to affect humans due to the various routes of transmission involved [28,29]. Recently, *L. garvieae* has been recognized as an opportunistic pathogen in humans, attributed to a rising incidence of infections that result in considerable morbidity and mortality [6,30,31,32,33]. Risk factors include exposure to or ingestion of raw fish, along with the presence of concurrent gastrointestinal disease. Even immunocompetent individuals could be susceptible when they consume undercooked fish [6,34]. The first human case of *L. garvieae* infection was reported in 1991 in a patient with endocarditis [28]. Bacterial endocarditis is one of the clinical manifestations, predominantly observed in elderly patients [29,35]. A limited number of cases of this rare condition has been reported globally, most notably in Europe, Turkey, North America, Latin America, and Asia [31,36,37,38,39,40,41,42,43,44,45,46,47,48,49,50,51]. Bacteremia and sepsis have been observed in both children and adults [33,50,51,52,53,54]. Other reported diseases associated with *L. garvieae* include infective endocarditis, septicemia, peritonitis, hepatic abscess, meningitis, tonsillitis, osteomyelitis, empyema, and urinary tract infections [6,42,47,49,55,56,57,58,59,60,61,62,63,64]. Also, the pathogen has been frequently isolated from stools [65]. In many patients infected with *L. garvieae*, the source of infections remains unknown [6,32,57].

Considering all of the above, this study aimed to test classical and molecular biology methods to identify *L. garvieae* and differentiate it from closely related species, perform epidemiological typing, and determine the antimicrobial resistance and factors of virulence of bacterial strains isolated in the period of 2002–2019 from various clinical specimens. It is crucial to identify the causative agent promptly and implement measures to restrict the dissemination of pathogens, thereby preventing outbreaks among fish, other species, and humans, as well as mitigating potential economic losses.

## 2. Materials and Methods

### 2.1. Sample Collection

A total of 24 strains were obtained over an eighteen-year period from 2002 to 2019, 21 (*n* = 21) of which were confirmed as *L. garvieae*. The other three were identified as *L. lactis*, *S. iniae*, and *E. faecalis* and subsequently used as negative controls. Isolates were collected from different cities in Bulgaria and Greece (Table 1). The specimens were from various organs of rainbow trout fish (*Oncorhynchus mykiss*) and Atlantic salmon (*Salmo salar*), including the heart, kidney, brain, small and large intestines, lungs, liver, and spleen.

### 2.2. L. garvieae Bacterial Isolates

*L. garvieae* was initially identified by colony and Gram staining morphology, alpha-hemolysis on blood agar, the positive pyrrolidonyl arylamidase (PYR) test, and negative catalase, oxidase, and motility tests. Strains were growth on Trypticase Soy Agar (TSA), Tryptic Soy Blood Agar (TSBA), Brain Heart Infusion Agar (BHIA), 5% sheep blood agar, and MRS agar. Growth in the presence of 6.5% NaCl and pH 9.6 on Brain Heart Infusion Broth (BHIB) was tested. Detailed biochemical identification was performed with BD BBL™ Crystal™ Gram-Positive (GP) (Becton, Dickinson and Company, Franklin Lakes, NJ, USA). Strains that correspond biochemically to the genus *Lactococcus* were additionally tested with Antony staining (for the demonstration of capsule production with India ink) and the slide agglutination test using specific anti-*Lactococcus garvieae* serum (NRL “FMCR”, NDRRIMI, Sofia, Bulgaria) against a capsular reference strain 331/8066/Belgium. The presumed identification of the species was conducted by MALDI-TOF MS (VITEK MS system) according to the manufacturer’s instructions (bioMerieux, Craponne, France).

The isolates were stored in tryptic soy broth (Sigma-Aldrich, Bengaluru, India) supplemented with 10% glycerin at −80 °C until the beginning of the tests. Prior to testing, they were subcultivated on tryptic soy blood agar (TSBA, Himedia, Thane, India) for 24 h at 37 °C under aerobic conditions.

*Lactococcus garvieae* ATCC 43921, confirmed isolates of *Lactococcus garvieae* from Serbia, *Streptococcus iniae*, *Lactococcus lactis*, and *Enterococcus faecalis* were used as positive and negative control strains in the experiments.

### 2.3. DNA Extraction

Pure cultures of all strains included in the study were used for genomic DNA extraction, which was performed using an extraction kit (NucleoSpin Tissue, Macherey-Nagel, Düren, Germany) in accordance with the manufacturer’s instructions. All extracted DNA was stored at −80 °C until testing

### 2.4. Antimicrobial Susceptibility Testing

Antimicrobial susceptibility testing was performed using the Kirby–Bauer disk diffusion method. Eight antibiotic disks were used to determine antimicrobial susceptibility: ampicillin (2 µg), ceftriaxone (30 µg), erythromycin (15 µg), tetracycline (30 µg), levofloxacin (5 µg), chloramphenicol (30 µg), vancomycin (5 µg), and linezolid (30 µg). Examination took place on Mueller Hinton Fastidious Agar (Horse blood 5% + 20 mg/L beta-NAD; Liofilchem, Roseto degli Abruzzi, Italy).

For interpretations of the results from antibiotic testing, EUCAST recommendations for Viridans group streptococci were used (EUCAST 2024) [66]. In the absence of criteria, the guidelines provided by the Clinical and Laboratory Standards Institute (CLSI) were applied [67].

### 2.5. Polymerase-Chain Reaction (PCR)

All collected strains were tested with PCR using forward and reverse primers, which amplify a fragment of 1100 bp, previously used for identification by Zlotkin et al. in 1998 [68]. A final confirmation test to differentiate *L. garvieae* from closely related species was conducted using primers that were previously described by Dang et al. in 2012 and Saticioglu et al. in 2023 [9,69] (Table 2). The MyTaq PCR mix was used (Bioline, Meridian Bioscience, Memphis, TN, USA). The reaction conditions for PCR were initial denaturation at 95 °C for 1 min followed by 35 cycles consisting of denaturation at 95 °C for 1 min, annealing for 45 sec, elongation at 72 °C for 1 min, and final elongation at 72 °C for 7 min. Gradient PCR with different annealing temperatures (53–63 °C) was performed in order to optimize the protocol of Zlotkin et al. [68] and to inhibit non-specific reactions [68]. The study used *Streptococcus iniae*, *Enterococcus fecalis*, and deionized water as negative controls.

The products before and after purification were controlled by gel electrophoresis in 2% agarose gel (Bioline, Meridian Bioscience, Memphis, TN, USA), 1× TAE buffer, a 10 pmol/mL concentration of ethidium bromide (Sigma-Aldrich, Steinheim, Germany), and DNA markers 100 bp (New England Biolabs, Ipswich, MA, USA) and 100 bp (Bioline, Meridian Bioscience, Memphis, TN, USA).

### 2.6. Sequencing of 16S rDNA Gene and Capsular Gene

The 16S rDNA gene was identified by sequencing using universal primers described by Lane et al. in 1991 (Table 2) [70]. The PCR conditions were similar to those described above. *L. lactis*, *S. iniae*, and *E. fecalis* were used as control strains.

To detect the presence of the 17 322 bp pathogenicity-associated island (capsule cluster) and a 750 bp capsule gene, the primers described by Miyauchi et al. in 2012 were used [71] (Table 2). The reaction was performed using the LongAmp Taq 2x Master Mix (New England Biolabs, Ipswich, MA, USA) and the following protocol: activation of polymerase for 30 s at 94 °C, followed by 30 cycles of denaturation at 94 °C for 30 s, annealing for 30 s, elongation at 65 °C for 3.5 min, and a final step at 65 °C for 10 min. The final products were prepared for sequencing by purification through NucleoSpin Gel and PCR Clean-up kit (Macherey-Nagel, Düren, Germany), according to the manufacturer’s instructions. The purified products were sequenced at Macrogen (Seoul, Republic of Korea). The obtained product was controlled as described above.

Obtained sequential data were processed and equalized by MEGA X software [72], using the MUSCLE algorithm [73], and were analyzed in BLAST (Basic Local Alignment Search Tool) NCBI (National Center for Biotechnology Information, U.S. National Library of Medicine, 8600 Rockville Pike, Bethesda, MD 20894, USA).

### 2.7. Epidemiological Typing by Random Amplification of Polymorphic DNA (RAPD Analysis)

Three primers were used to perform RAPD analysis: E1 (CCCAAGGTCC) [74], P2 (GTTTCGCTCC) [75], and RAPD 4 (AAGACGCCGT) [76]. Reactions with individual primers were performed in volumes of 50 µL including 4 µL of DNA, 4 µL of primer, 25 µL of the Kapa Ready PCR mix (KapaBiosystems), and 17 µL of deionized water. Two programs were tested with the following temperature parameters:

1st variant: activation of polymerase at 95 °C for 1 min, followed by 45 cycles: denaturation at 95 °C for 1 min, annealing at 36 °C for 1 min (modification from Ravelo et al., 54/2003 (35 °C) [75]), and elongation at 72 °C for 2 min.

2nd variant: activation of polymerase at 94 °C for 5 min, followed by: 4 cycles at 94 °C—45 s, 30 °C—2 min, and 72 °C—30 s; 10 cycles at 94 °C—45 s, 36 °C—30 s, 72 °C—30 s; 10 cycles at 94 °C—45 s, 36 °C—30 s, 72 °C—40 s; 10 cycles at 94 °C—45 s, 36 °C—30 s, 72 °C—40 s; 10 cycles at 94 °C—45 s, 36 °C—30 s, 72 °C—50 s; 10 cycles at 94 °C—45 s, 36 °C—30 s, 72 °C—1 min; final extension at 72 °C for 10 min.

The resulting fragments from the reactions with the three primers were compared visually in 2% agarose gel with 1× TAE buffer and 10 pmol/mL ethidium bromide (Sigma-Aldrich, Steinheim, Germany), and subsequent phylogenetic analysis was performed.

The photo documentation system “Syngene GelVue” model No. GVM20 (Synoptics Ltd., Cambridge, UK) was used.

### 2.8. Data Analysis

The following programs were used to analyze the data:

The software GeneTools v. 4.1 (ChemiGenius, Syngene, Cambridge, UK) was used for the similarity matrices calculations on the basis of the electrophoretic gel images and the construction of the unweighted pair group method with arithmetic (UPGMA) dendrograms using the “profile” and “band position” options. Calculations were performed by means of the Jaccard algorithm [77], accounting not only for their positions but the peak surfaces of the fluorescent bands as well.

The evolutionary history of the 16s rDNA gene was inferred by using the Maximum Likelihood method and the Kimura 2-parameter model [78]. The tree with the highest log likelihood (−3008.43) is shown. The percentage of trees in which the associated taxa clustered together is shown next to the branches. Initial tree(s) for the heuristic search were obtained automatically by applying the Neighbor-Joining and BioNJ algorithms to a matrix of pairwise distances estimated using the Maximum Composite Likelihood (MCL) approach and then selecting the topology with the superior log likelihood value. The bootstrap 1000 replicates were used. A discrete Gamma distribution was used to model evolutionary rate differences among sites (5 categories (+G, parameter = 0.5289)). The proportion of sites where at least 1 unambiguous base is present in at least 1 sequence for each descendent clade is shown next to each internal node in the tree. Evolutionary analyses were conducted in MEGA X [72].

## 3. Results

### 3.1. Cultivation and Identification

The cultural characteristics of the isolates presented small, rounded shapes with smooth edges and a convex profile when grown on MRS agar. They demonstrated α-hemolysis on blood agar. Biochemical analysis revealed that they were catalase-negative and -positive for the PYR reaction. Gram and Anthony staining procedures were performed, revealing the presence of Gram-positive ovoid cocci, occurring either singly or in groups of 2–3 cells, as well as in short chains (Supplementary Figure S1) with capsules (Supplementary Figure S2).

All tested isolates showed positive agglutination with rabbit anti-*L. garvieae* serum, except for strains 472 and 332 (Supplementary Figure S3).

Further biochemical identification was conducted with BD BBL™ Crystal™ Gram-positive (GP) (Becton, Dickinson and Company, USA). Nineteen strains were identified as *L. garvieae*. *L. garvieae* strains in the BBL Crystal Gram-positive ID System showed variability in 2 tests: sucrose and 4-methyl umbelliferyl-N-acethyl-b-D-glucosaminide (FGA) in combinations S+/FGA− and S−/FGA+.

Using MALDI-TOF MS spectrophotometric analysis, two more strains with positive agglutination were identified as *L. garvieae*. Isolate 472 was identified as *L. lactis*, isolate 330 as *S. iniae*, and isolate 332 as *E. faecalis*.

### 3.2. Antimicrobial Susceptibility

All *L. garvieae* strains were resistant to clindamycin and susceptible to ampicillin, ceftriaxone, erythromycin, tetracycline, levofloxacin, chloramphenicol, vancomycin, and linezolid.

### 3.3. Molecular-Genetic Methods

**Figure 1:**
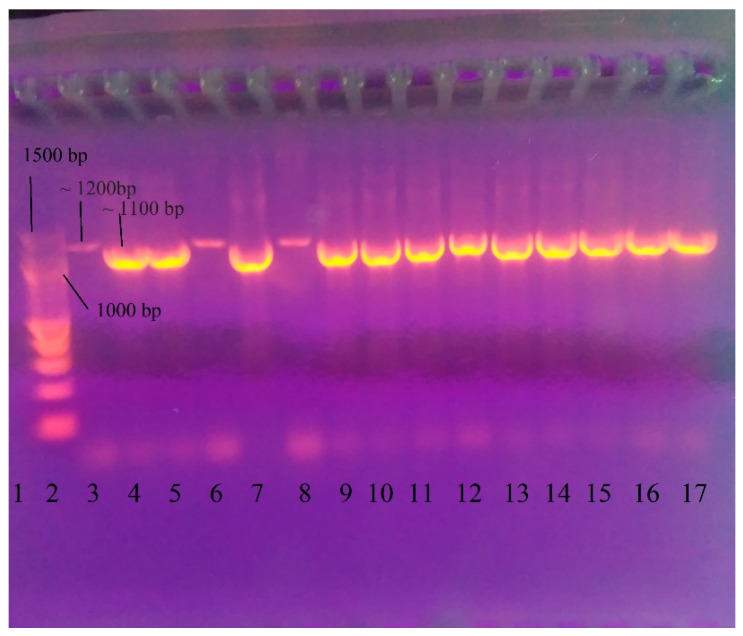
Gel electrophoresis after PCR for identification of *L. garvieae* using the amplification protocol of Zlotkin et al. [68]. 1—negative control (double distilled water); 2—DNA Ladder 100 bp (New England Biolabs, Ipswichm, MA, USA); 3—472 *L. lactis* (~1200 bp); 4—331 *L. garvieae* (~1100 bp); 5—412 *L. garvieae*; 6—330 *Streptococcus iniae* (~1200 bp); 7—443 *L. garvieae*; 8—332 *Enterococcus faecalis* (~1200 bp); 9—17 *L. garvieae* isolates—322, 329, 386, 400, 418, 443, 459, 467, and 470.

After DNA had been extracted from the strains, all DNA was tested by PCR to detect *L. garvieae* using the protocol of Zlotkin et al. [68]. The gel electrophoresis demonstrated the presence of PCR products not only from *L. garvieae* but also from the other three isolates that tested negative for *L. garvieae* (*S. iniae*, *Enterococcus faecalis*, and *L. lactis*). However, there were minor differences in both fragment length and signal intensity (Figure 1).

**Figure 2:**
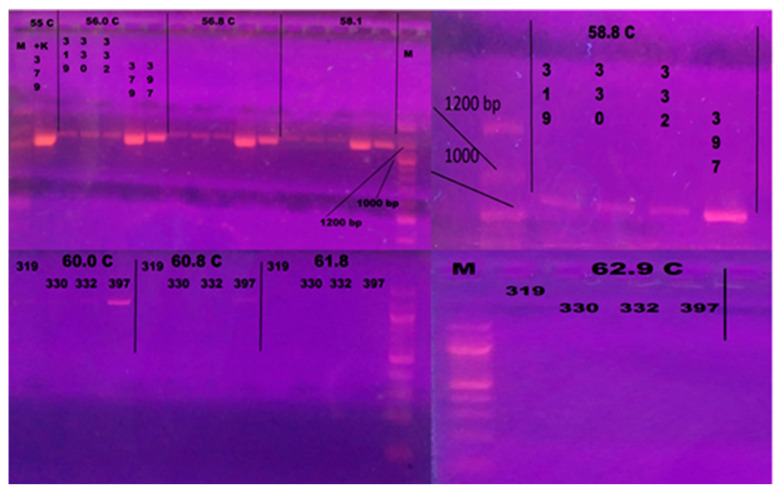
Gel electrophoresis after gradient PCR for optimization of annealing temperature. The following isolates were included: 319—*L. lactis*; 330—*Streptococcus iniae*, 332—*Enterococcus faecalis*; 379 *L. garvieae*; 397—*L. garvieae*. M—100 bp Ladder (New England Biolabs, Ipswich, MA, USA). Annealing temperatures of 60.8 °C and 61.8 °C resulted in products specific to *L. garvieae*. The lower temperature (60.8 °C) was selected for the optimized protocol.

The results of PCR performed following the protocol of Zlotkin et al. [68] showed that the method needed optimization [68]. Gradient PCR was conducted using a range of annealing temperatures from 55 °C to 62.9 °C to optimize the protocol and remove products from strains that were not *L. garvieae*. Annealing temperatures of 60.8 °C and 61.8 °C resulted only in *L. garvieae* products (Figure 2). The lower temperature (60.8 °C) was selected for the optimized protocol.

A final confirmation test for differentiation from closely related species in the genus *Lactococcus* was performed. PCR with primers LG_IBS_F (132), LG_IBS_R (132), LP_IBS_F (583), LG_IBS_R (583), ITS 30F (290), and ITS 319R (290) showed that 21 isolates were *L. garvieae* [9,69].

**Figure 3:**
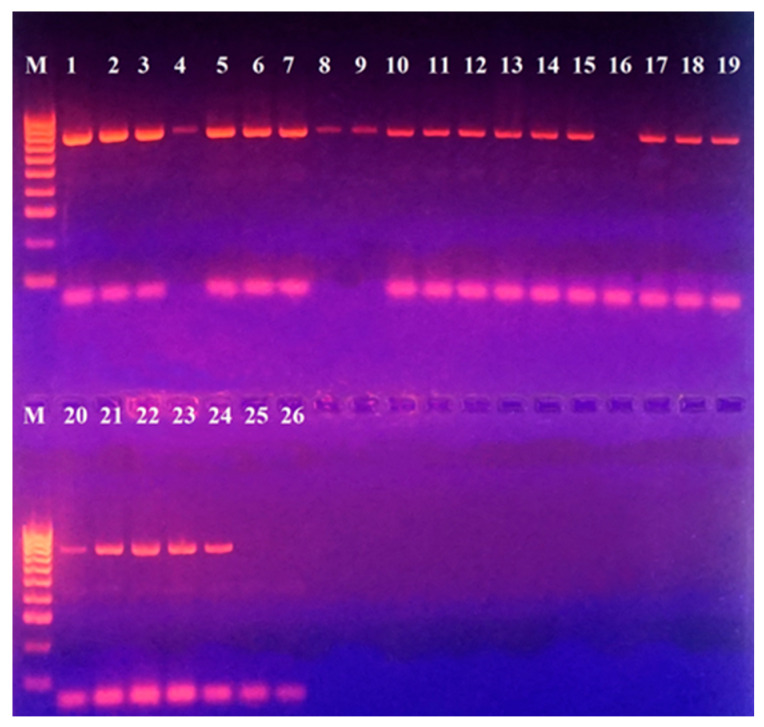
PCR for detection of a pathogenicity island with included capsule production gene in *L. garvieae* strains included in this study. M—DNA Ladder 100 bp (Bio-line, Meridian Bioscience, TN, USA); Positions: 1—331; 2—322; 3—386; 4—329; 5—397; 6—400; 7—415; 8—412; 9—465; 10—417; 11—418; 12—443; 13—456; 14—459; 15—466; 16—468; 17—467; 18—469; 19—470; 20—471; 21—473 Serbian control *L. garvieae*; 22—474 Serbian control *L. garvieae*; 23—442; 24—444; 25—472 *L. lactis*; 26—Negative control (double-distilled water).

PCR for the identification of a 17,322 bp pathogenicity island (capsular cluster) was performed in *L. garvieae* strains and no such pathogenicity island was found. All of the strains, except for 468, demonstrated the presence of a ~750 bp product (Figure 3).

**Figure 4:**
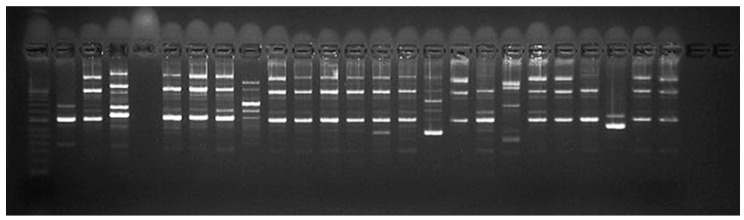
Gel electrophoresis after epidemiological typing with primer P2; Position 1—Control ladder; Position 2—331/2002/Belgium; Position 3—322/2002/Dospat; Position 4—negative control; Position 5—386/2006/Dospat; Position 6—397/2008/Dospat; Position 7—400/2008/Dospat; Position 8—412/2010/Dospat; Position 9—415/2010/Greece; Position 10—417/2013 Dospat; Position 11—418/2013/Dospat; Position 12—443/2016/salmon; Position 13—456/2017/Dospat; Position 14—459/2017/Dospat; Position 15—465 brain of a cooled trout bought in 2017 from a fish stall at ‘Krasno selo’ market; Position 16—466 brain of a cooled trout bought in 2017 from a fish stall at supermarket; Position 17—467 heart of a trout from a fish farm in the town of Pirdop, 2019; Position 19—469 brain of a trout before therapy, town of Pirdop, 2019; Position 20—470 spleen of a trout, town of Pirdop, 2019; Position 21—471 after florfenicole treatment, town of Pirdop, 2019; Position 22—472 town of Pirdop, 2019; Position 24—473/2019/Serbian control *L. garvieae*; Position 25—474/2019/Serbian control *L. garvieae*.

**Figure 5:**
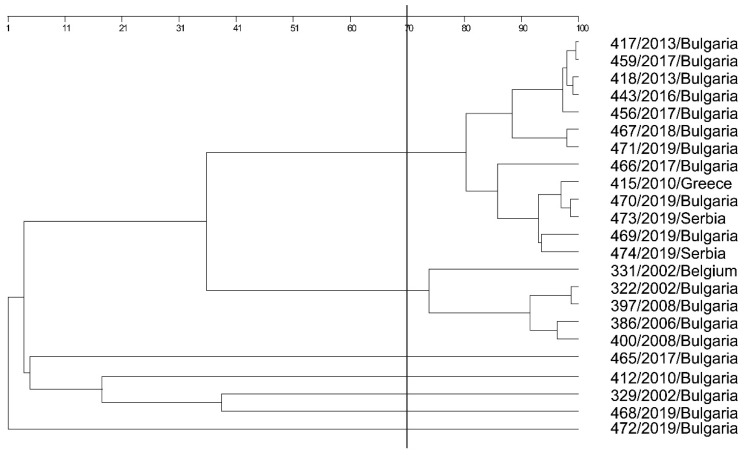
Phylogenetic analysis (epidemiological typing) based on primer P2 and 23 *L. garvieae* strains, and a single *L. lactis* strain—472/2019.

Epidemiological typing with both programs and three primers showed the largest number of fragments with primers P2 and E1. Typing with the primer RAPD4 showed too high homogeneity of the results in most strains (Figure 4 and Figure S4). These results were also reflected in the dendrogram based on the resulting fragments. Accordingly, the most appropriate primer for epidemiological typing was selected based on the position of strain *L. lactis* (472) included in this analysis. Namely, the selected primer was P2, which positioned it as an external group (Figure 5).

The results of capsular gene sequencing were not suitable for processing due to background noise. Electropherograms from the 16S rRNA gene showed no background noise and were processed.

**Figure 6:**
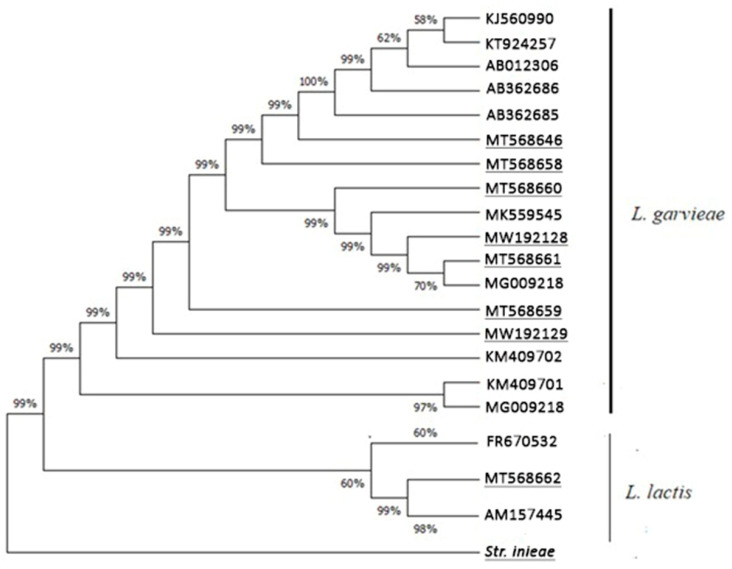
The evolutionary history. This analysis involved 21 nucleotide sequences with standard 27F и 1492R primers for 16s rDNA gene. Sequences from our isolates (underlined) and the NCBI database were used. As an external group, *S. iniae* was used. There were a total of 1294 positions in the final dataset. Data were visualized via MEGA X [72].

Multiple alignments of processed sequential data were performed, and *L. garvieae* sequences were chosen from GeneBank (NCBI). The most appropriate model for phylogenetic analysis was described by Kimura et al. [78]. The sequenced 16s rRNA genes of the included strains were processed and analyzed (Blast-NCBI) and showed 100% identity to the six *L. garvieae* strains stored in GeneBank (NCBI). A phylogenetic analysis was performed (Figure 6).

## 4. Discussion

Lactococcosis is a bacterial disease that usually presents as acute hemorrhagic septicemia in fish, causing morbidity and mortality at rates between 20% and 50%. Infections have been reported in a wide range of hosts—fish, reptiles, marine mammals, cows, dogs, birds, and humans [6]. *L. garvieae* is considered the most important pathogen associated with lactococcosis. This microorganism is considered an emerging opportunistic agent, affecting primarily immunosuppressed individuals [30,31,32,33]. Genera with similar cultural and biochemical characteristics, as well as closely related species (*Lactococcus petauri* and *Lactococcus formosensis*), additionally complicate microbiology diagnosis, which requires a combination of morphological, physiological, and genetic methods [3].

The morphology of strains used in the current study corresponds to the typical features of *L. garvieae* described by other authors [9,79,80]. The colonies exhibited α-hemolysis on blood agar, which is generally more common than β-hemolysis observed in atypical isolates from water buffalo mastitis [13,75,80,81].

All *L. garvieae* isolates exhibited negative catalase activity and positive PYR tests; however, these tests are insufficient to differentiate them from enterococci. BD BBL™ Crystal™ Gram-positive (GP) (Becton, Dickinson and Company, USA) misdiagnosed two of the strains, while MALDI-TOF identified them as *L. garvieae*.

On the one hand, some authors have applied the method of Zlotkin et al. [68] to identify and/or confirm *L. garvieae* in clinical specimens from humans and fish, without detecting amplification in other bacterial species [2,24,38,39,40,68,75]. On the other hand, non-specific reactions, yet with small differences in the fragment length (100 bp), have been found by other authors [13]. In our study, positive results from other species (*L. lactis*, *E. faecalis*, and *S. iniae*) were found when the annealing temperature was up to 60 °C. Both different sizes and weaker signal intensities due to various product quantities were observed. These contradictory data about the presence or absence of non-specific reactions in the same microorganisms are possibly due to various factors such as strain specifications, method execution, types and sources of the reagents (whether the Taq polymerase is hot start or not), and the operator. It is well known that higher annealing temperatures may increase the specificity of PCR, which is the basis for optimizing the protocol and eliminating the non-specific reaction with the previously mentioned microorganisms, thus improving the diagnostic power of the method. This is useful in cases where no controls from other bacterial species are loaded as markers for non-specific reactions [11]. In this study, a combination of methods was suggested to eliminate the disadvantages of the Zlotkin protocol [68].

Antimicrobial susceptibility testing of lactococci was conducted in accordance with the established criteria for Viridans group streptococci. The decision was based on the fact that they both belong to the same family of *Streptococcaceae*, as well as on their common cultural characteristics. All examined isolates were resistant to clindamycin. This feature was used to differentiate *L. lactis* from *L. garvieae* [28].

Some authors divide *L. garvieae* serologically into three groups based on geographical differences, while others divide them into two groups, but both classifications have KG− (capsular) and KG+ (non-encapsulated), with KG− being more virulent [82,83,84]. When testing for the presence of a pathogenicity island, the presence of a cluster was not detected, but the presence of a capsule gene was observed in three of the isolates, showing poor capsule production and one isolate lacking a capsule gene. This result showed that with the exception of a single KG+ isolate, all other strains belonged to the European capsular serotype KG−, meaning that highly virulent strains were predominant in our study [1,84].

The results from capsular gene sequencing were not suitable for processing due to the presence of too many signaling peaks. These cannot be identified as ‘background noise’, but as rather overlapping peaks, which could be caused by various factors during the PCR processes, purification, and sequencing or the gene being non-homogeneous in the bacterial population [85]. However, as the same bacterial cultures were used and the results from 16s rDNA gene sequences were indisputable, the most probable reason was a non-homogeneous gene. The weaker visualization of the capsular gene on the agarose gel was likely due to the non-homogeneous bacterial population. Some bacterial cells may produce capsules while others may not. Such heterogeneity of the bacterial population with regard to the capsular gene was (to some extent) confirmed by the capsular staining performed where various-size capsules were observed in pure bacterial culture (the same cultures were used in other methods in this study).

Ravelo et al. tested a series of primers (P1–P6) for RAPD analysis of *L. garvieae* from various hosts (yellowtail, rainbow trout, and catfish) and various geographic regions and determined primers P5 and P6 as the most appropriate for both features—geographic origin and host [75]. The resulting fragments were from 7 up to 14. In this study, *L. garvieae* strains were collected from rainbow trout and Atlantic salmon. *L. lactis* was used as an external group control. Therefore, tests were conducted on alternative primers—E1, P2, and RAPD4—using different amplification protocols. Variant II of our program demonstrated the best representation (5–12 fragments) with primer P2. The difference between our results and those of Ravelo et al. was most likely due to the different amplification programs, as the authors used 30 amplification cycles with the same parameters: 95 °C–35 °C–72 °C in the different steps [75]. The program is very similar to variant I described in the Section 2. In our study, the annealing temperature was 36 °C, as higher temperatures may increase the reaction specificity, which indicates that a greater number of DNA fragments is likely to be present at lower temperatures [85].

Some authors applied other primers for RAPD, such as M13 and P1–P6 [11,13,75]. Similarly, the P2 primer in our study ensured a sufficient number of fragments for the subsequent phylogenetic analysis of the epidemiological typing. The closely related *L. lactis*, which provided non-specific PCR cross-reactions with *L. garvieae*, was well distinguished in epidemiological typing, so the method is appropriate to differentiate those two species.

Using a closely related species as an external group in epidemiological typing is an important index for the specificity of the PCR products. This is important because, afterward, the studied species will be differentiated from each other based on the external group—the root of the tree. *L. lactis* was precisely positioned as an external group in the phylogenetic analysis of *L. garvieae* strains. Moreover, by using the P2 primer, information about the genetic and phenotypic changes in the species through the years was gained. It followed a logical pattern: two major groups were formed based on the strain history and similarity confidence threshold (SCT) 70. Strains 468, 329, 412, and 465 were individual, separate from all other strains, and the closest to the external group (*L. lactis*), yet these could not be defined as separate branches (SCT < 40). Strain 468/2019 was the closest to the external group. As several strains were isolated from the same farm, the *L. lactis* (472) strain was isolated after therapy against *L. garvieae*. Strain 468 was non-capsular (like 472 *L. lactis*). In addition, all three dendrograms showed that 468 was a separate branch. This is an indication that the method using the P2 primer includes the region of the capsular gene and carries important information about the strain’s virulence. Strain 329 was isolated from fish brains; the clinical manifestation was mild, as was the production of capsular polysaccharides (Figure 6). Strain 412/2010 was a vaccinal strain from the Greek clinical isolate 415/2010. The last strain (465/2017) from this group was isolated from a rainbow trout purchased from a store; the fish exhibited no pathological changes in its internal organs. Thus, it can be concluded that a separate group of non-virulent strains was formed. All other strains were clinical (except for strain 466), capsular, and highly virulent and formed two major groups (SCT > 70).

Strain 466/2010 was additionally isolated from a retail store located in a different region, yet, unlike strain 465, it was in the group of clinically high virulent capsular isolates.

These results show that the methodology for epidemiological typing of *L. garvieae* strains by using P2 was informative and suitable for prognoses and studies.

Sequenced 16s rDNA of the included strains were processed and analyzed in Blast-NCBI and showed 100% identity with *L. garvieae* sequences deposited in NCBI. Thus, 16s rDNA sequencing analysis confirmed the microbiological, biochemical, and molecular identification of the isolated bacteria as belonging to the species *L. garvieae*. The phylogenetic analysis conducted formed groups of *L. lactis* and *L. garvieae*; the latter was represented by two major branches. The phylogenetic analysis based on 16s defined our isolates in an *L. garvieae* group together with isolates from India, South Africa, and Japan, thus confirming the identification as *L. garvieae* [86,87]. Some authors [32,88] applied phylogenetic analysis based on high-sensitivity methods, such as detecting a housekeeping gene or whole-genome sequencing. In contrast, the use of only 16s provides limited epidemiological information and is suitable as a complementary method.

In recent years, new species closely related to *Lactococcus garvieae* have been described that cannot be discriminated using MALDI-TOF, 16S sequencing, or biochemical tests [3,7]. This is why new approaches have been developed to identify these species: whole-genome sequencing, intergenic spacer 16S–23S sequencing, *gyrB* gene sequencing, multiplex PCR, and serotype analysis using PCR [7,8,9,10]. In the current study, the final identification of *L. garvieae* was performed using multiplex PCR as described by Dang et al. and Saticioglu et al. [9,69].

The strains included in this study belonged to the European capsular and non-capsular (strain 468) types. Epidemiological typing with the P2 primer formed groups of less virulent (non-capsular or weak capsule producers) and capsulated virulent strains. Moreover, the virulent capsular strains were grouped into two major branches, showing a change in the *L. garvieae* genome over the years: namely, group I comprised the period of 2002–2008 and group II included the period of 2013–2019. In group II, a relationship between a Greek clinical strain from 2010 and clinical strains from Bulgaria and Serbia isolated in 2019 was confirmed. This is proof that genetically similar strains are circulating in the Balkan region, which may be associated with the commercial exchange of breeding fish and ready-to-use products.

## 5. Conclusions

Due to morphological, phenotypical, and genetic similarities between different species of cocci and among the representatives of the genus *Lactococcus*, a combination of methods is necessary to discriminate *L. garvieae*. Its importance is linked to possible outbreaks in fish and other animals, which can cause significant economic losses and possible transmission to immunocompromised patients acting as an opportunistic agent. Additionally, further investigation into the genetic basis of the observed virulence and resistance patterns, especially regarding the capsular gene region identified through the RAPD method, could provide deeper insight into the pathogenicity of *L. garvieae*. Such studies could also explore the potential for targeted prophylaxis based on the specific virulence factors of the strains. Future research should encompass a broader range of fish species, particularly those that are commonly consumed by humans.

## Figures and Tables

**Table 1:** Strains of *L. garvieae* and other bacterial species included in the study.

Laboratory No./NCBI Number 16S rDNA	Year of Collection	Origin	Species
331/8066/MT568646	2002	Belgium/reference strain	*L. garvieae*
322	2002	Dospat town/RTF	*L. garvieae*
329	2002	Dospat town/RTF	*L. garvieae*
379	2006	Dospat town/RTF	*L. garvieae*
a386	2006	Dospat town/RTF	*L. garvieae*
397	2008	Dospat town/RTF	*L. garvieae*
400	2008	Dospat town/RTF	*L. garvieae*
412/MT568658	2010	Greece/RTF	*L. garvieae*
415	2010	Greece/RTF	*L. garvieae*
417	2013	Dospat town/RTF	*L. garvieae*
418	2013	Dospat town/RTF	*L. garvieae*
443 MT568659	2016	Bulgaria/AS	*L. garvieae*
444	2016	Bulgaria/AS	*L. garvieae*
456	2017	Dospat town/RTF	*L. garvieae*
459	2017	Dospat town/RTF	*L. garvieae*
465	2017	Sofia city/from store/RTF	*L. garvieae*
466	2017	Sofia city/from store/RTF	*L. garvieae*
467/MT568660	2019	Pirdop town/RTF	*L. garvieae*
468	2019	Pirdop town/RTF	*L. garvieae*
469	2019	Pirdop town/RTF	*L. garvieae*
470	2019	Pirdop town/RTF	*L. garvieae*
471	2019	Pirdop town/RTF	*L. garvieae*
472/MT568662	2019	Pirdop town	*L. lactis*
473		Serbia/control	*L. garvieae*
474		Serbia/control	*L. garvieae*
330	2019	Sofia city	*Str. iniae*
332	2018	Sofia city	*Ent. faecalis*

RTF—rainbow trout fish (*Oncorhynchus mykiss*); AS—Atlantic salmon (*Salmo salar*).

**Table 2:** Primer sequences and amplification conditions for PCR of genes used in the current study.

	Primer Sequence (5′→3′)	Product Size (bp)	Annealing Temperature (°C)	Reference
pLG-1 pLG-2	CATAACAATGAGAATCGC GCACCCTCGCGGGTTG		60.8 *	[68]
LG_IBS_F(132) LG_IBS_R(132)	ACAGAGCATGGGACGACCTA CTGCTTCTTGAGAGACGCCA		54	[9]
LP_IBS_F(583) LG_IBS_R(583)	ATTGGCTTAGGGGTTTGGGG AGTCCGAAATACGTTCCCGG		54	[9]
ITS 30F(290) ITS 319R(290)	ACTTTATTCAGTTTTGAGGGGTCT TTTAAAAGAATTCGCAGCTTTACA		54	[69]
27F 1492R	AGAGTTTGATCCTGGCTCAG TACGGYTACCTTGTTACGACTT		56	[70]
Lg2F Lg2R	TGCTGTCATCATATTGTGTCCA GGCTATGGCATTAGTCAGGAAG		55	[71]

* Optimized annealing temperature using gradient PCR.

## Data Availability

The original contributions presented in this study are included in the article and Supplementary Materials. Further inquiries can be directed to the corresponding author.

## Supplementary Materials

The following supporting information can be downloaded at: https://www.mdpi.com/article/10.3390/microorganisms13020436/s1, Supplementary File S1 with four Figures S1–S4. Figure S1: Gram stain method of isolate 443; Figure S2: Anthony stain method for capsule of isolate 443; Figure S3: Gruber type agglutination reaction with specific rabbit anti-*L. garvieae* serum against capsular isolate 443; Figure S4: Gel electrophoresis after epidemiological typing with primers: Ist row (top)—E1; IInd row (middle)—P2; and III row (bottom)—RAPD4; Position 1—Control ladder; Position 2—331/2002/Belgium; Position 3—322/2002/Dospat; Position 4—negative control; Position 5—386/2006/Dospat; Position 6—397/2008/Dospat; Position 7—400/2008/Dospat; Position 8—412/2010/Dospat; Position 9—415/2010/Greece; Position 10—417/2013 Dospat; Position 11—418/2013/Dospat; Position 12—443/2016/salmon; Position 13—456/2017/Dospat; Position 14—459/2017/Dospat; Position 15—465 brain of a cooled trout bought in 2017 from a fish stall at “Krasno selo”market; Position 16—466 brain of a cooled trout bought in 2017 from a fish stall at “Fantastiko” supermarket; Position 17—467 heart of a trout from a fish farm in the town of Pirdop, 2019; Position 19—469 brain of a trout before therapy, town of Pirdop, 2019; Position 20—470 spleen of a trout, town of Pirdop, 2019; Position 21—471 after florfenicole treatment, town of Pirdop, 2019; Position 22—472 town of Pirdop, 2019; Position 23—473/2019/Serbian control *L. garvieae*; Position 24—474/2019/Serbian control *L. garvieae*.
