# Supplementary material for: Identification, Characterization, and Epidemiological Analysis of Lactococcus garvieae Fish Isolates Obtained in a Period of Eighteen Years
Source: Microorganisms. 2025 Feb 17;13(2):436. doi: 10.3390/microorganisms13020436 (PMC11858575; doi:10.3390/microorganisms13020436)
Supplement: Supplementary file 1 [file microorganisms-13-00436-s001.zip › microorganisms-3480314-supplementary.pdf]

Supplementary file

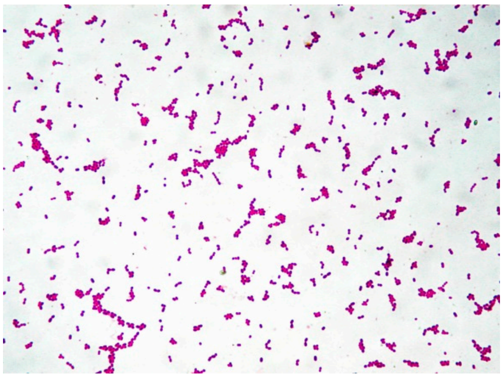

Supplementary Figure S1. Gram stain method of isolate 443**.**

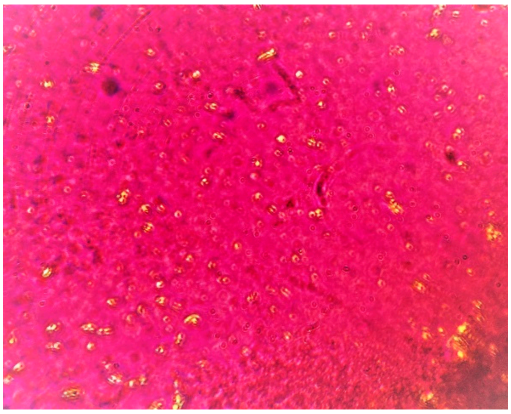

Supplementary Figure S2. Anthony stain method for capsule of isolate 443.

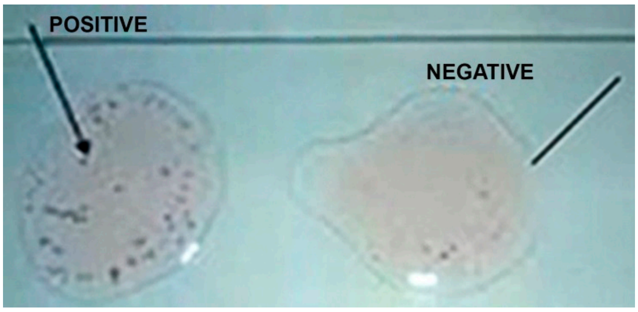

Supplementary Figure S3. Gruber type agglutination reaction with specific rabbit anti-*L.garvieae* serum against capsular isolate 443.

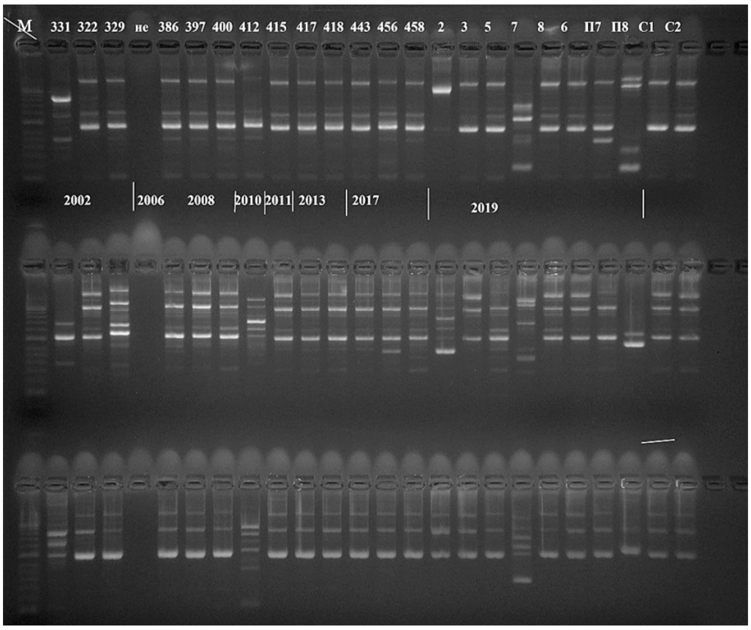

Supplementary Figure S4. Gel electrophoresis after epidemiological typing with primers: Ist row (top) - E1; IInd row (middle) - P2; and III row (bottom) - RAPD4; Position 1 – Control ladder; Position 2 – 331/2002/Belgium; Position 3 – 322/2002/Dospat; Position 4 – negative control; Position 5 – 386/2006/Dospat; Position 6 – 397/2008/Dospat; Position 7 – 400/2008/Dospat; Position 8 – 412/2010/Dospat; Position 9 – 415/2010/Greece; Position 10 – 417/2013 Dospat; Position 11 – 418/2013/ Dospat; Position 12 – 443/2016/salmon; Position 13 – 456/2017/Dospat; Position 14 – 459/2017/Dospat ; Position 15 – 465 brain of a cooled trout bought in 2017 from a fish stall at "Krasno selo"market; Position 16 – 466 brain of a cooled trout bought in 2017 from a fish stall at "Fantastiko" supermarket; Position 17 – 467 heart of a trout from a fish farm in the town of Pirdop, 2019; Position 19 – 469 brain of a trout before therapy, town of Pirdop, 2019; Position 20 – 470 spleen of a trout, town of Pirdop, 2019; Position 21– 471 after florfenicole treatment, town of Pirdop, 2019; Position 22 – 472 town of Pirdop, 2019; Position 23 – 473/2019/Serbian control *L. garvieae*; Position 24 – 474 /2019/Serbian control *L. garvieae*.
